# Bee Venom Alleviates Motor Deficits and Modulates the Transfer of Cortical Information through the Basal Ganglia in Rat Models of Parkinson’s Disease

**DOI:** 10.1371/journal.pone.0142838

**Published:** 2015-11-16

**Authors:** Nicolas Maurice, Thierry Deltheil, Christophe Melon, Bertrand Degos, Christiane Mourre, Marianne Amalric, Lydia Kerkerian-Le Goff

**Affiliations:** 1 Aix Marseille Université, CNRS, IBDM UMR 7288, Marseille, France; 2 Aix Marseille Université, CNRS, LNC UMR 7291, Marseille, France; 3 INSERM, CNRS, Collège de France, CIRB UMR 7241 U-1050, Paris, France; 4 APHP, Département des Maladies du Système Nerveux, Centre Expert Inter-Régional Ile de France de la Maladie de Parkinson, Hôpital Pitié-Salpêtrière, Paris, France; Florey Institute of Neuroscience & Mental Health, AUSTRALIA

## Abstract

Recent evidence points to a neuroprotective action of bee venom on nigral dopamine neurons in animal models of Parkinson’s disease (PD). Here we examined whether bee venom also displays a symptomatic action by acting on the pathological functioning of the basal ganglia in rat PD models. Bee venom effects were assessed by combining motor behavior analyses and *in vivo* electrophysiological recordings in the substantia nigra pars reticulata (SNr, basal ganglia output structure) in pharmacological (neuroleptic treatment) and lesional (unilateral intranigral 6-hydroxydopamine injection) PD models. In the hemi-parkinsonian 6-hydroxydopamine lesion model, subchronic bee venom treatment significantly alleviates contralateral forelimb akinesia and apomorphine-induced rotations. Moreover, a single injection of bee venom reverses haloperidol-induced catalepsy, a pharmacological model reminiscent of parkinsonian akinetic deficit. This effect is mimicked by apamin, a blocker of small conductance Ca2+-activated K+ (SK) channels, and blocked by CyPPA, a positive modulator of these channels, suggesting the involvement of SK channels in the bee venom antiparkinsonian action. *In vivo* electrophysiological recordings in the substantia nigra pars reticulata (basal ganglia output structure) showed no significant effect of BV on the mean neuronal discharge frequency or pathological bursting activity. In contrast, analyses of the neuronal responses evoked by motor cortex stimulation show that bee venom reverses the 6-OHDA- and neuroleptic-induced biases in the influence exerted by the direct inhibitory and indirect excitatory striatonigral circuits. These data provide the first evidence for a beneficial action of bee venom on the pathological functioning of the cortico-basal ganglia circuits underlying motor PD symptoms with potential relevance to the symptomatic treatment of this disease.

## Introduction

In Parkinson’s disease (PD), the neurodegeneration of the dopaminergic neurons of the substantia nigra pars compacta (SNc) leads to severe motor deficits linked to profound alterations in the functioning of the cortico-basal ganglia-thalamo-cortical loop circuits [1,2]. The adverse effects resulting from the long-term dopamine replacement pharmacotherapy using L-DOPA has fostered the research of alternative or adjunctive symptomatic treatments, including pharmacological, surgical and cell- or gene-based therapies [3–6]. Neuroprotective strategies targeting main PD pathogenic mechanisms are also explored [7,8]. Identifying agents combining symptomatic and disease-modifying potential is therefore of primary interest.

Evidence has been provided for a neuroprotective action of bee venom (BV) both *in vitro* on cell cultures and *in vivo* in animal models of neurodegenerative diseases, including mouse models of PD [9–15]. Apamin, one BV component, is a blocker of small conductance Ca^2+^-activated K^+^ (KCa2 or SK) channels that have been shown to modulate dopamine neuron survival or phenotype [9,16,17] and are attracting increasing interest for pharmacotherapy [18–20]. Confirming the interest of targeting SK channels in PD, apamin has been recently reported to reinstate minimal DA activity in the striatum and alleviate the non-motor symptoms induced by partial DA lesions [21]. Apamin also alleviated motor symptoms in extensive dopamine degeneration model [21], suggesting that the effects of SK channel blockade can bypass the dopamine system.

This study is the first to evaluate the symptomatic action of BV and its impact on the pathological functioning of the basal ganglia in rat models of PD. We demonstrated that BV treatment efficiently alleviated akinesia-like deficit in both the neuroleptic-induced catalepsy model and in the hemiparkinsonian rat model based on extensive 6-hydroxydopamine (6-OHDA)-induced lesion of nigral dopamine neurons. We then investigated the cellular substrates of BV action in these models by *in vivo* electrophysiological recordings of spontaneous and cortically-evoked neuronal discharge patterns in the substantia nigra pars reticulata (SNr) the main basal ganglia output structure in rodents. The results showed that BV does not act by normalizing the pathological firing of SNr neurons, but by modulating the transfer of cortical information through the basal ganglia.

## Material and Methods

Animal experimental procedures were carried out in strict accordance with the recommendations of the European Communities Council Directive (2010/63/EU) and conformed to the ethical guidelines of the French Ministry of Agriculture and Forests. Experimental procedures were approved by the local Institutional Animal Care and Use Committee (IBDM SBEA, Structure chargée du bien-être des animaux de l'Institut de Biologie du Développement de Marseille) and were authorized by the Animal Health and Protection Veterinary Service (authorization #13–400 granted to Nicolas Maurice). Male Wistar rats (160–180 g, Charles River Laboratories, L'Arbresle, France) were maintained on a 12:12-h light/dark cycle and temperature-controlled conditions (21 ± 2°C), with food and water ad libitum.

### Drugs

Bee venom (*Apis mellifera)*, apamin (SK channel blocker), CyPPA (cyclohexyl-[2-(3,5-dimethyl-pyrazol-1-yl)-6-methyl-pyrimidin-4-yl]-amine; SK channel positive modulator), haloperidol (preferential dopamine D2 receptor antagonist), raclopride (dopamine D2 receptor antagonist), SCH23390 (R(+)-7-chloro-8-hydroxy-3-methyl-1-phenyl-2,3,4,5,-tetrahydro-1H-3-benzazepine; dopamine D1 receptor antagonist) and 6-OHDA were purchased from Sigma-Aldrich (Lyon, France). Drugs were prepared as follows: haloperidol in distilled water with a methylparaben and propylparaben solution and a drop of lactic acid (0.1 N), CyPPA in saline containing ethanol 0.1%, SCH23390 in distilled water and the other drugs in saline, added with 0.1% ascorbic acid for 6-OHDA and apomorphine.

### 6-OHDA lesion, pharmacological treatments and control of the lesion extent

Animals under equithesin anesthesia (4 ml/kg) received 6 μl of 6-OHDA hydrochloride solution (2 μg/ μl) or vehicle at 1 μl/min rate in the left SNc (stereotaxic coordinates from interaural according to De Groot [22]: A: 2.2 mm, L: 2.0 mm, DV: 3.3 mm, incisor bar +5.0 mm).

**Fig 1:**
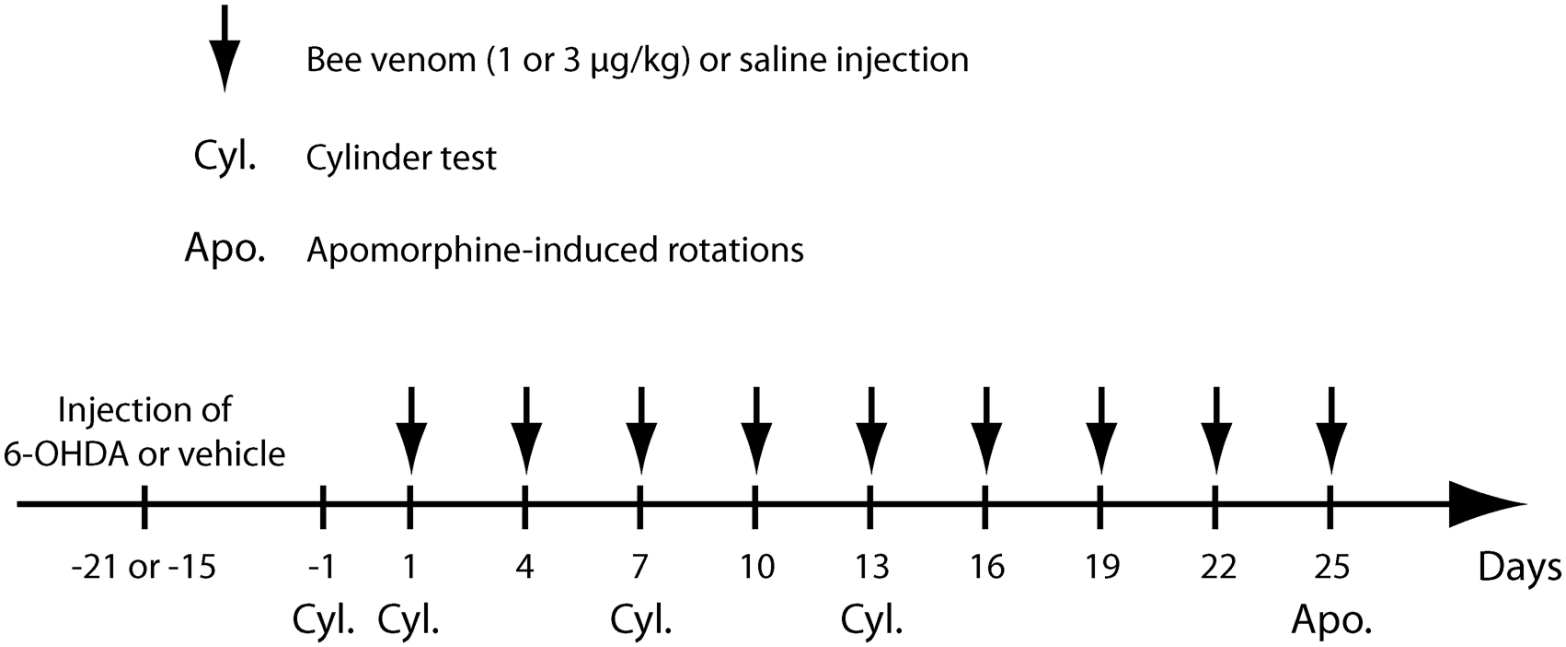
Time schedule for bee venom injections and behavioral testing in 6-OHDA lesioned rats. Time points at which 6-OHDA lesion and bee venom (1 μg/kg for BV1 group or 3 μg/kg for BV3 group) or saline i.p. injections were carried out. Note that bee venom or saline was given every 3 days, starting 15–21 days after the 6-OHDA injection. Cyl. and Apo. indicate time points at which rats were taken for the cylinder test (Cyl.) or apomorphine-induced rotations (Apo.), respectively. After the apomorphine-induced rotations test, all rats were killed for brain processing and analysis.

As illustrated in Fig 1, at 15–21 days post-surgery, animals used for behavioral analyses received BV at 1 or 3 μg/kg, i.p. (BV1 and BV3 groups) or saline every 3 days. The cylinder test was performed the day prior to first injection and then at the first, third and fifth injection. Apomorphine-induced rotation was measured at the 9^th^ BV injection (day 40 or 46 post-surgery). For electrophysiological studies, animals received a single BV injection (3μg/kg,ip) during the recording session.

At completion of the experiments, striatal dopamine denervation extent was assessed by analysis of [^3^H]-mazindol binding to DA uptake sites on film autoradiograms [23]. Only the animals showing extensive loss of dopamine terminals in the striatum ipsilateral versus contralateral to the lesion (n = 46) were kept for data analysis (means: -95.1 ± 1.7% in dorsal striatum and -73.2 ± 5.6% in ventral striatum).

### Behavioral analyses

#### Cylinder test

6-OHDA-lesioned rats were scored for contralateral forelimb akinesia in the cylinder test [24], 40 min after BV or vehicle injection. Rats were placed in a Plexiglas cylinder (19 cm diameter, 30 cm high) and videotaped for 10 min. The results are the number of contacts made on the cylinder wall with both paws (double contacts) expressed as percentage of the total number of contacts (double + single contacts). Data are means ± SEM of values determined from 5–7 animals per group. Statistical comparison was performed using two-way repeated measures ANOVA with treatment (saline, BV1 and BV3) as the between-subject factor and repeated injections as the within-subject factor followed by multiple comparisons versus pre-treatment (Holm-Sidak method).

#### Apomorphine-induced turning behavior

Rotations (full 360° turns) induced by apomorphine (0.1 mg/kg s.c.) in lesioned rats were recorded for 70 min using automated rotameter cylinders (TSE, Bad Homburg, Germany). Comparisons were made between saline and BV3 group (n = 5/group). BV was injected 30 min prior apomorphine injection. Results were expressed as mean net rotations (contra-ipsi) per 5 min and as total net rotations over the recording period. Data were analyzed by two-way repeated measures ANOVA with groups as between-subject factor and time as within subject factor followed by Newman-Keuls tests for comparison at each time point. Comparison of total net rotations used unpaired t-test.

#### Haloperidol-induced catalepsy

Animals received haloperidol (1 mg/kg i.p.) either alone or followed 30 min later by injection of BV, apamin, CyPPA or co-injection of BV+CyPPA or apamin+CyPPA (n = 6–8 animals per group). BV (1 or 3 μg/kg) and apamin (0.1, 0.2, or 0.4 mg/kg) were administered by i.p. injections 30 min before test. CyPPA was infused intracerebroventricularly in behaving animals previously implanted with a guide cannula positioned 1 mm above the lateral ventricle one week before testing. The surgical conditions were similar to the 6-OHDA lesions with the addition of the implantation of screws fixed with dental cement to maintain the guide cannula. The day of testing, an injection cannula (30 gauge) was lowered through the guide (23 gauge) so that its tip reached the ventricle (AP -0.9 mm; L ± 1.8 mm from bregma; DV– 2.2 mm from bregma, with the incisor bar set at -3.3 mm [25] and CyPPA infused by gravity at the dose of 0.5μg/μl in a volume of 2 μl just before test. In the co-treatment conditions, CyPPA was tested against doses of BV or apamin providing the greatest anti-cataleptic effects (3 μg/kg for BV and 0.1 mg/kg for apamin). Catalepsy was assessed using the bar test. The latency (in seconds; cut-off time, 120 s) to step down a rod suspended 9 cm above the floor was measured every 30 min from 60 to 240 min after haloperidol injection. Typically, control animals with no haloperidol injection did not maintain their position on the bar after three attempts and remained no more than 1 or 2 sec on the bar at each measure. They were considered as non-cataleptic. Median latency to step down the bar was determined from 6–8 animals per group. Statistical comparisons were performed using Kruskal-Wallis one-way ANOVA on ranks followed by multiple comparisons versus haloperidol alone group (Dunn’s Method).

### Electrophysiological recordings

Animals under chloral hydrate anesthesia [400 mg/kg, i.p. for induction, supplemented by continuous delivery by a peristaltic pump (Prosciences, Paris) at 60 mg/kg/h, i.p.] were fixed in a stereotaxic head frame (Horsley-Clarke apparatus; Unimécanique, Epinay-sur-Seine, France), and body temperature maintained at 36.5°C.

#### Firing activity

**Fig 2:**
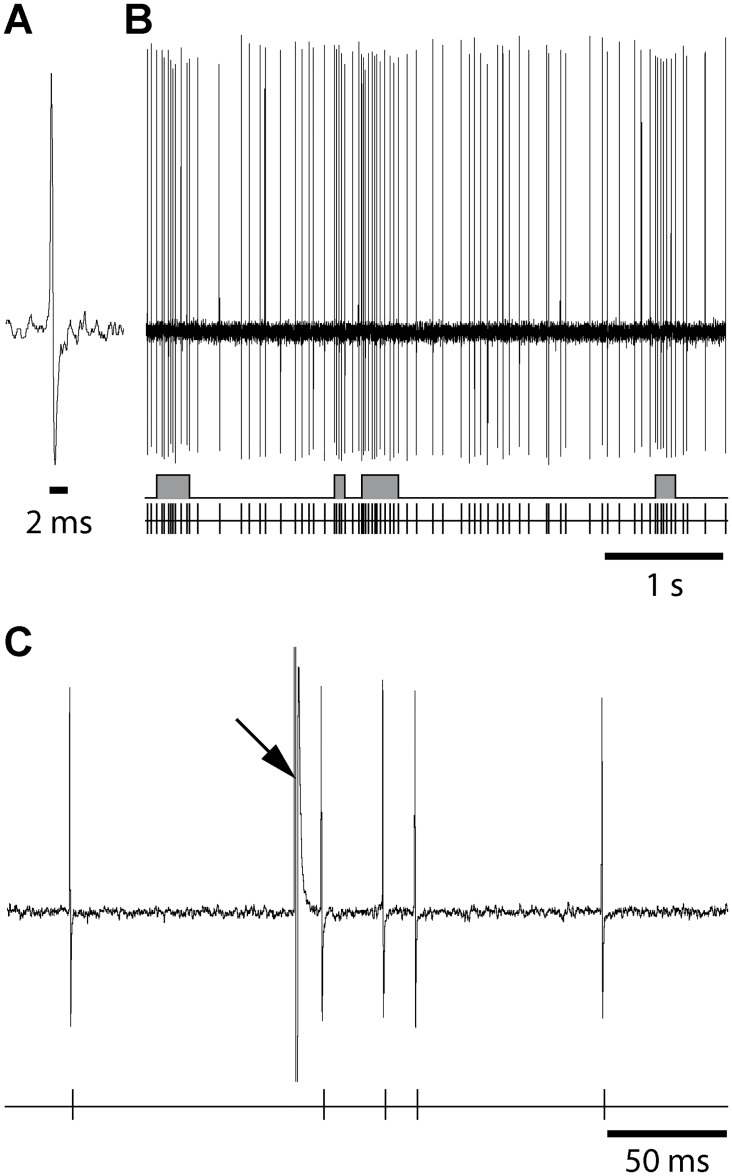
Raw recording traces from a SNr cell illustrating the short spike duration (*A*), its spontaneous discharge (*B*) and its response to one single orofacial motor cortex stimulation (*C*). (***A***) Magnified view of recording traces from a SNr neuron illustrating that its spikes are narrow (< 2 ms). (***B***) The spontaneous activity is displayed following injection of haloperidol; the discharge of the cell is represented as the raw recording trace (top), the result of the Poisson Surprise spike analysis (S ≥ 2) indicating bursts (middle) and the corresponding sequence of spikes (bottom). (***C***) Response evoked by a single cortical stimulation illustrated as a raw trace (top) and the corresponding sequence of spikes (bottom). Arrow indicates the artifact stimulation.

Single-unit activity of neurons in the lateral division of the SNr (A, 3.0–3.4 mm; L, 2.3–2.6 mm; DV, 6.8–7.8 mm), which receives inputs from the sensorimotor cortex [26], was recorded extracellularly [27] using glass micropipettes (15-20MΩ) filled with a 0.5 M NaCl solution containing 1.5% Neurobiotin (Vector Laboratories, Burlingame, CA). Action potentials were recorded using an Axoclamp-2B amplifier (Molecular Devices, Union City, CA), amplified, filtered with an AC/DC amplifier (DAM 50; World Precision Instruments, Stevenage, UK) and displayed on a digital oscilloscope (TD 3014 B; Tektronix, Courtaboeuf, France). Data were sampled on-line at 10 kHz rate on a computer connected to a CED 1401 interface and off-line analyzed using Spike 2 program (Cambridge Electronic Design, Cambridge, UK). Nigral neurons were identified as non-dopaminergic by their classically defined electrophysiological characteristics: narrow spikes (width ≤ 2 ms), as illustrated in Fig 2A, and ability to present relatively high-frequency discharges (> 10 Hz) without decrease in spike amplitude [28,29]. For discharge pattern analysis, spontaneous activity was analyzed by periods of 150 s. Epochs of elevated discharge rate were classified as bursts using a Poisson Surprise analysis [30,31]. This was done using a script written for the Spike2 software. Briefly, this analysis evaluates how improbable any given burst, that contains n spikes in a time interval T, occurred by chance and computed as follows: S = -log *p*, where *p* is the probability that, in a random (Poisson) spike train having the same average spikes rate r as the spike train studied, a given time interval of length T contains n or more spikes. *p* is given by Poisson's formula, as follows:

$$p\;=\;e^{-rT}\overset{\infty }{\underset{i=n}{\sum }}{\left(rT\right)}^{i}/i!$$

where S refers to the Poisson Surprise of the burst (the degree to which the burst surprises a person who expects the spike train to be a Poisson process). In this study, only spike trains with S ≥ 2 were considered to be bursts (Fig 2B).

#### Cortical stimulation-evoked responses

The orofacial motor cortex [A (from interaural): 12.5 mm; L: 3.8 mm; DV (from cortical surface): 1.2 mm; [25]] ipsilateral to the recorded SNr was stimulated through bipolar coaxial stainless steel electrodes (SNE-100; Rhodes Medical Instruments, Woodlands Hill, CA). Stimulations consisted of pulses of 600 μs width and 10–20 V, delivered at 1 Hz. Peristimulus time histograms were generated from 50 stimulation trials. Spikes were discriminated from noise and stimulation artifacts based on their amplitude (Fig 2B), using the gate function of the 121 window discriminator (World Precision Instruments), and sampled on-line. Cortical stimulation evoked complex responses in SNr cells, consisting in most cases of a sequence of excitation-inhibition-excitation, respectively attributed to activation of the “hyperdirect” trans-subthalamic pathway, the “direct” and the “indirect” trans-striatal pathways [27,32]. Excitatory responses were defined as > 50% increase vs. pre-stimulus in the number of spikes for at least three consecutive bins and were characterized by their latency, duration and amplitude. Inhibitory responses corresponded to the time interval during which no spike was observed, and were characterized by their latency and duration. It is to note that variations in anesthesia level are unlikely to account for changes in cortically-evoked responses of SNr. On the one hand, these responses are elicited by electrical shocks that strongly synchronize the cortex, overpassing any other cortical rhythm. Accordingly, triphasic responses are steadily observed under different anesthetics[33,34] or even in awake state as shown in monkey[35] and in human[36]. On the other hand, the response of a given cell to a given cortical stimulation is very reliable over time[34].

#### Data analysis

Numerical values are given as means ± SEM. Statistical analysis was performed using SigmaPlot 12.5 (Systat software Inc., San Jose, CA) and significance was assessed by performing appropriate statistical tests (paired Student's t test or, for multiple comparisons, one-way repeated measure ANOVA followed by Holm-Sidak post-hoc test).

## Results

### Bee venom impact on haloperidol-induced catalepsy

**Fig 3:**
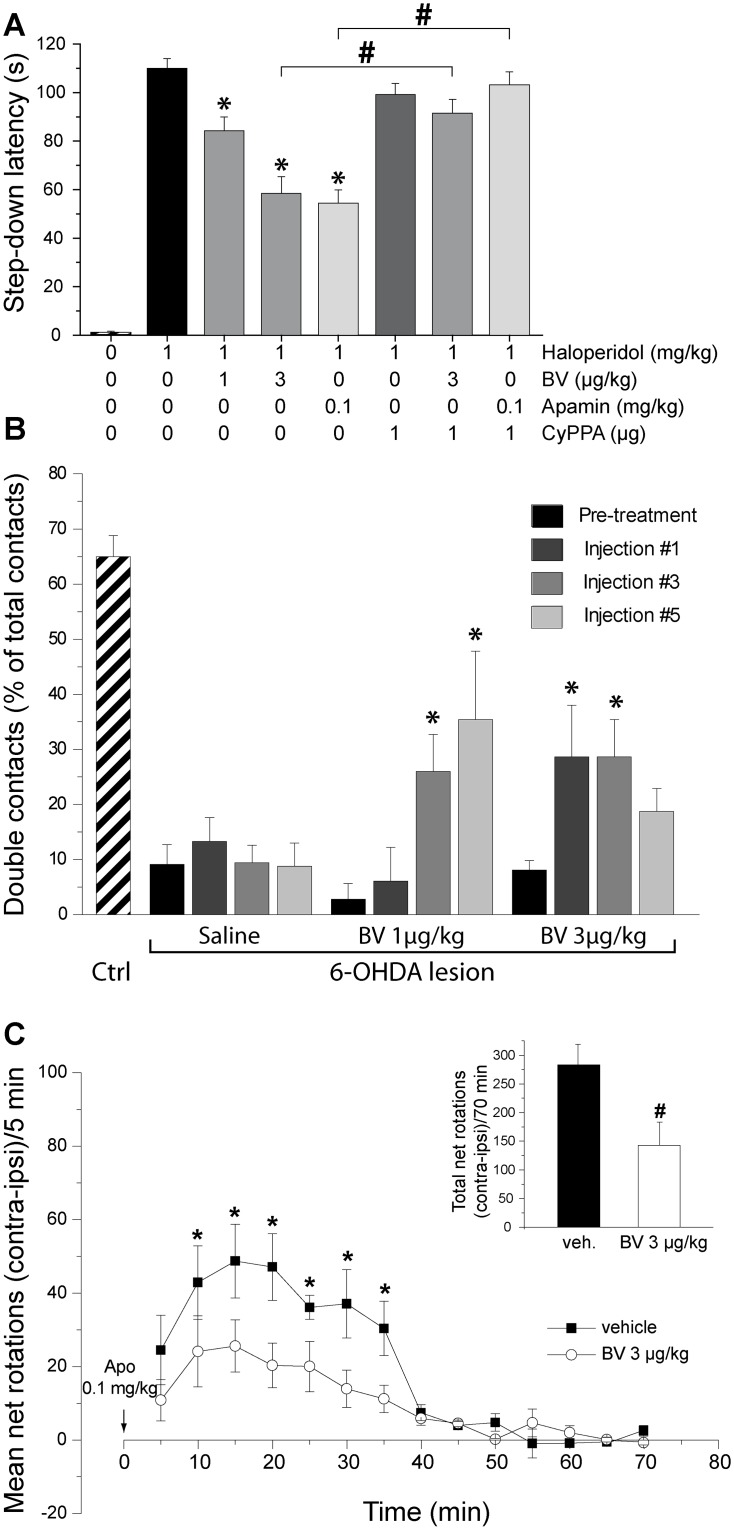
Bee venom alleviates parkinsonian-like deficits. (***A***) Effects of bee venom and apamin on haloperidol-induced catalepsy in rats. Rats received a single injection of haloperidol (1 mg/kg i.p.). After 30 min, animals received either an i.p. administration of BV (1, and 3 μg/kg; n = 7/group) or apamin (0.1 mg/kg; n = 8/group) in combination or not with CyPPA (1 μg, i.c.v.). Haloperidol-injected animals receiving vehicle served as reference. Catalepsy was measured 30 min later for 120 min. The histograms represent the mean latencies to step down the rod ± S.E.M. during the 120-min test. * *p* < 0.05 vs. haloperidol + vehicle, Kruskal-Wallis one-way ANOVA followed by multiple comparisons versus control group (Dunn's Method). # *p* < 0.01, Mann-Whitney Rank Sum Test. (***B***) Effects of acute and repeated bee venom injections on 6-OHDA lesion-induced forelimb akinesia evaluated using the cylinder test. The data presented in the histogram are the means ± SEM of the numbers of double contacts made with the forepaws on the wall of the cylinder, expressed as the percentage of the total number of contacts, determined from n animals. Statistical comparison was performed using two-way repeated measures ANOVA with treatment (saline, BV 1 μg/kg and BV 3 μg/kg) as the between-subject factor and repetitions of injections as within subject factor followed by multiple comparisons versus pre-treatment (Holm-Sidak method) with an overall significance level of 0.05. * Statistically different from pre-treatment score. The numbers of animals per group were as follows: controls = 7, 6-OHDA + Saline = 7, 6-OHDA + BV 1 μg/kg = 5 and 6-OHDA + BV 3 μg/kg = 7. (***C***) Effects of bee venom on apomorphine-induced circling behavior. Unilateral 6-OHDA-lesioned rats were treated with vehicle or BV (9 injections, i.p, n = 5/group), and 30 min after the last injection they received apomorphine (0.1 mg/kg s.c.). The total numbers of ipsilateral and contralateral rotations were measured for 70 min immediately after apomorphine injection. The graphs represent the means ± S.E.M. of net rotations (number of contralateral minus ipsilateral rotations) for every 5-min interval. The histograms (inset) represent the means ± S.E.M. of the total net rotations for the 70 min period. * *p* < 0.01 vs. vehicle group for time, two-way repeated measures ANOVA; # *p* < 0.05 vs. vehicle group, t-test.

Haloperidol produced a profound cataleptic state as shown by the high median latency to step down from the bar compared to control animals that could not remain on the bar (Fig 3A). Kruskal-Wallis one-way ANOVA showed significant difference among the treatment groups (P<0.001). Acute administration of BV or of apamin significantly reduced this latency. CyPPA, which had no effect on its own, prevented the anti-cataleptic action of BV and of apamin (Fig 3A).

### Bee venom effect on motor deficits in hemiparkinsonian rats

#### Cylinder test (Fig 3B)

Sham-operated rats (n = 7) made a majority of double contacts (65.0 ± 3.8%) on the cylinder wall. Before treatment, the proportion of double contacts in hemiparkinsonian rats from the saline, BV1 and BV3 groups fell to 9.1 ± 4.4%, 2.8 ± 5.2% and 8.1 ± 4.4% respectively, due to decreased spontaneous use of the forepaw contralateral to the lesion. Statistical comparisons showed significant effect of repeated drug administration (F_3,6_ = 6,413; *p* < 0.001) and treatment/repetition interaction (F_6,48_ = 4,332; *p* < 0.002). Compared to pre-treatment, the BV1 group showed significant improvement of the motor score at the third and fifth injections but not at the first injection. The BV3 group showed significant recovery at the first and third injection while the effect of the fifth injection was not significant.

#### Apomorphine-induced circling (Fig 3C)

BV (3 μg/kg) significantly reduced apomorphine-induced contralateral rotations (treatment effect: F_1,8_ = 6.784, *p* < 0.05; treatment x time: F_13,104_ = 2.408, *p* < 0.01). Total number of apomorphine-induced rotations decreased from 283.3 ± 35.7 to 142.9 ± 40.4 (*p* = 0.031, Student t-test; Fig 3C, inset).

### Effects of haloperidol on the spontaneous discharge of SNr neurons and bee venom impact

The firing properties of SNr cells were analyzed repeatedly in control condition and 60 min after haloperidol injection alone or followed 30 min later by BV injection. In the haloperidol alone group (n = 14 animals), 7 out of the 43 cells recorded in the control period, which could be monitored long enough to measure the effects of haloperidol on their basal activity, were used for pairwise analyses. The results are shown in Table 1A. In the control period, these SNr cells exhibited a regular firing, with a low burst occurrence and limited proportion of spikes contributing to bursting activity. After haloperidol injection, the mean firing frequency was not significantly modified (*p* = 0.734; paired t-test), but the discharge pattern was altered. The mean recurrence of bursts tended to increase, although not significant (*p* = 0.055, paired t-test), and the percentage of spikes contributing to bursts was significantly increased (*p* = 0.015, paired t-test).

**Table 1:** Characteristics of the spontaneous discharge of SNr cells according to the different treatments or bee venom injection.

***A—In control condition and after systemic injection of haloperidol***			
N = 7	Discharge rate (Hz)	Mean recurrence of bursts (in bursts/min)	Percentage of spikes contributing to burst activity (%)
Control	21.6 ± 3.7	9.9 ± 4.6	8.4 ± 3.6
Haloperidol Alone	20.7 ± 4.5	22.5 ± 7.6	31.2 ± 9.7*^1^
***B—In control condition and after systemic injection of haloperidol+bee venom***			
N = 7	Discharge rate (Hz)	Mean recurrence of bursts (in bursts/min)	Percentage of spikes contributing to burst activity (%)
Control	16.2 ± 2.4	15.9 ± 6.2	15.7 ± 6.1
haloperidol + BV	29.3 ± 6.9	42.5 ± 12.2	32.3 ± 5.5*^2^
***C—In control condition and after 6-OHDA lesion***			
N = 14	Discharge rate (Hz)	Mean recurrence of bursts (in bursts/min)	Percentage of spikes contributing to burst activity (%)
Control	19.5 ± 2.1	13.7 ± 4.6	11.3 ± 3.4
6-OHDA	18.3 ± 3.1	24.3 ± 4.6*^3^	28.9 ± 5.7*^4^
***D—In 6-OHDA lesioned animal and after systemic injection of bee venom***			
N = 8	Discharge rate (Hz)	Mean recurrence of bursts (in bursts/min)	Percentage of spikes contributing to burst activity (%)
6-OHDA	21.3 ± 6.4	23.4 ± 4.7	22.0 ± 5.8
6-OHDA + BV	15.0 ± 4.6	17.8 ± 4.6	33.2 ±10.3

Each cell was recorded successively in the two conditions indicated in the first column.

*^1^ to *^4^: statistically different from the pre-treatment value; paired t-test; ***A***: *^1^ *p* = 0.015; ***B***: *^2^ *p* = 0.036; ***C***: *^3^ *p* = 0.002; *^4^ *p* = 0.012.

In the haloperidol+BV (3 μg/kg) group (n = 11 animals), 7 out of the 35 cells recorded in the control period could be kept for monitoring after BV and were used for pairwise analyses. The characteristics of the discharge vs. control period were affected similarly to that observed in the haloperidol alone group (Table 1B): no change in the mean firing frequency (*p* = 0.14, paired t-test), tendency for increased burst recurrence that did not reach significance (*p* = 0.071, paired t-test) and increased percentage of spikes contributing to bursts (*p* = 0.036, paired t-test). This indicates that BV does not interfere with the effects of haloperidol on the spontaneous discharge properties of SNr neurons.

### Bee venom impact on neuroleptic-induced alterations of cortically-evoked responses in SNr neurons

**Fig 4:**
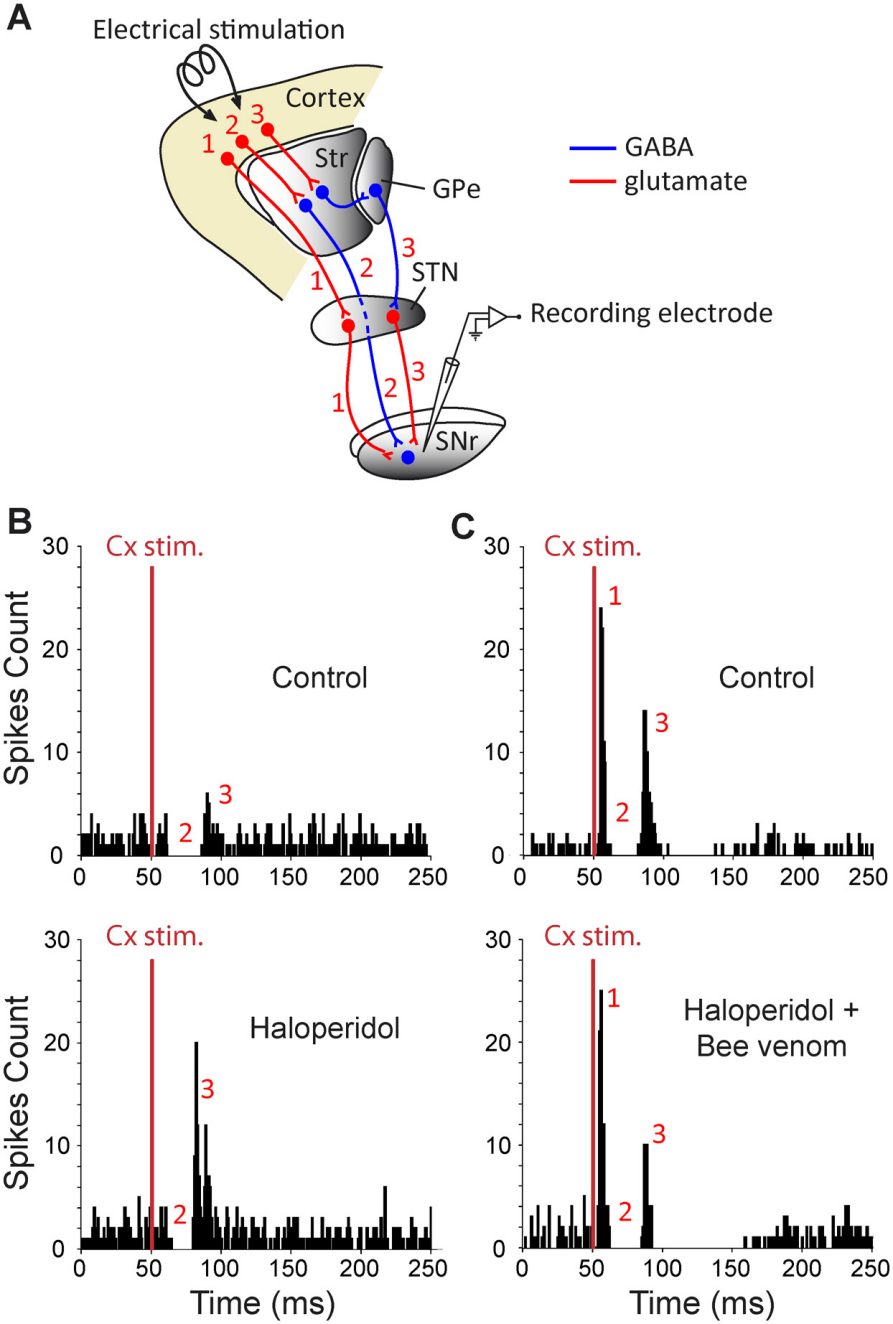
Experimental design (*A*) and effect of systemic injection of haloperidol alone (*B*) or followed by bee venom injection (*C*) on cortically-evoked responses on SNr neurons. (***A***) Schematic representation illustrating the three main basal ganglia pathways activated by cortical stimulation and connecting the cerebral cortex to the SNr. It includes the hyperdirect cortico-subthalamo-nigral pathway (#1) and the direct (#2) and the indirect (#3) striato-nigral pathways. Str, striatum; GPe, external globus pallidus; STN, subthalamic nucleus. (***B*, *top***) In this SNr neuron, orofacial sensori-motor cortex stimulation in control conditions evoked a complex response composed of an inhibition followed by a late excitation. (***B*, *bottom***) 60 minutes after haloperidol injection (1 mg/kg), the late excitation of the cortically-evoked response was markedly increased. (***C*, *top***) Classical triphasic excitatory-inhibitory-excitatory sequence evoked by stimulation of the orofacial sensori-motor cortex in control conditions in another SNr cell. In this cell, the increase of the late excitatory component was prevented by injecting BV 30 minutes after haloperidol as shown by comparing the cortical responses elicited 60 minutes after haloperidol (***C*, *bottom***) to the control response of the same SNr cell (***C*, *top***). Red numbers in ***B***-***C*** indicate which pathway in A is responsible for each component (excitation-inhibition-excitation) of the cortically-evoked responses. The same number of cortical stimulations (red bar, n = 50) was applied in ***B-C***.

The transfer of cortical information through the basal ganglia, assessed by recording the responses evoked by motor cortex stimulation in single SNr neuron, was analyzed using the same experimental paradigm (repeated recordings in control condition and after haloperidol treatment, alone or followed by BV 3μg/kg) (Fig 4). The cortically-evoked responses can vary among cells but remain stable in a given cell. Therefore, comparisons were made for a same cell under different successive conditions and not between cells from different experimental groups.

In the haloperidol alone group, 7 SNr neurons showing triphasic response to cortical stimulation were successively recorded in control and treatment periods (Table 2A and Fig 4A). The early excitation and following inhibition parameters were not significantly affected 60 minutes after haloperidol injection. By contrast, the late excitation amplitude was markedly increased (+108%; *p* = 0.016; paired t-test). In the neurons (n = 7) recorded in the haloperidol+BV group, there was no more alteration in the late excitation (Table 2B) versus in the control period and the other parameters were again unchanged (Fig 4B). These results indicate that BV prevents the haloperidol-induced changes in the transfer of cortical information through the indirect trans-striatal pathway, without altering the unaffected transfer through the hyperdirect and direct pathways.

**Table 2:** Characteristics of the responses evoked on the same SNr cell by electrical stimulation of the motor cortex in the different conditions.

***A—In control condition and after systemic injection of haloperidol***								
	Early excitation			Inhibition		Late excitation		
N = 7	L (ms)	D (ms)	Nb Sp	L (ms)	D (ms)	L (ms)	D (ms)	Nb Sp
Control	7.4±1.6	6.6±1.0	18.7±6.7	17.1±2.1	16.6±2.7	29.2±6.0	12.0±3.4	19.3±4.7
Haloperidol Alone	6.6±1.0	7.6±0.8	18.0±7.1	18.0±1.9	21.4±5.3	32.5±1.6	12.0±2.0	40.3±7.1* ^**1**^
***B—In control condition and after systemic injection of haloperidol + bee venom***								
	Early excitation			Inhibition		Late excitation		
N = 7	L (ms)	D (ms)	Nb Sp	L (ms)	D (ms)	L (ms)	D (ms)	Nb Sp
Control	6.3±0.7	6.3±0.7	34.0±8.9	15.6±0.7	19.1± 0.7	36.1±0.8	7.9±1.3	59.5±23.1
haloperidol + BV	5.8±0.6	7.6±1.1	44.7±14.5	14.4±0.9	20.0±1.1	35.7±1.5	8.4±0.8	58.3±27.3
***C—In control condition*, *after systemic injection of neuroleptics and after systemic injection of bee venom***								
	Early excitation			Inhibition		Late excitation		
N = 7	L (ms)	D (ms)	Nb Sp	L (ms)	D (ms)	L (ms)	D (ms)	Nb Sp
Control	9.0±0.4	6.6± 0.9	16.8±2.9	17.9±0.9	13.3±1.8	31.1±2.3	15.1±2.4	27.6±5.3
SCH 23390 + Raclopride	9.6±0.8	7.1±0.7	17.3±5.0	21.0±1.5	4.0±1.0* ^**2**^	25.6±1.8* ^**3**^	19.9±1.3* ^**4**^	47.6±12.7
SCH 23390 + Raclopride + BV	10.7±1.4	5.6±0.6	20.1±3.7	18.7±1.0	10.7±1.3	29.4±0.9	13.8±1.9	50.7±13.1
***D—In 6-OHDA lesioned animal and after systemic injection of bee venom***								
	Early excitation			Inhibition		Late excitation		
N = 8	L (ms)	D (ms)	Nb Sp	L (ms)	D (ms)	L (ms)	D (ms)	Nb Sp
6-OHDA	7.0±0.9	8.2±1.2	19.5±5.6	16.1±1.1	18.7±1.3	34.1±3.2	18.2±2.5	38.7±11.9
6-OHDA + BV	7.6±1.1	7.7±1.5	19.0±5.9	16.2±1.1	25.1±2.4* ^**5**^	34.3±3.1	19.1±2.9	39.2±12.8

The latency (L) and duration (D) of the different components of the cortically-evoked response were measured on the basis of post-stimulus time histograms generated from 50 cortical stimulations. Nb Sp: number of spikes.

***A***: *^1^ *p* = 0.016, Paired t-test; ***B***: N.S., Paired t-test; ***C***: *^2^: *p* < 0.001, One way Repeated Measures Analysis of Variance; *^3^: *p* = 0.027, One way Repeated Measures Analysis of Variance; *^4^: *p* = 0.021, One way Repeated Measures Analysis of Variance (Holm-Sidak Method for all RM ANOVA); ***D***: *^5^ *p* = 0.012, Paired t-test. Nb Sp: number of spikes.

**Fig 5:**
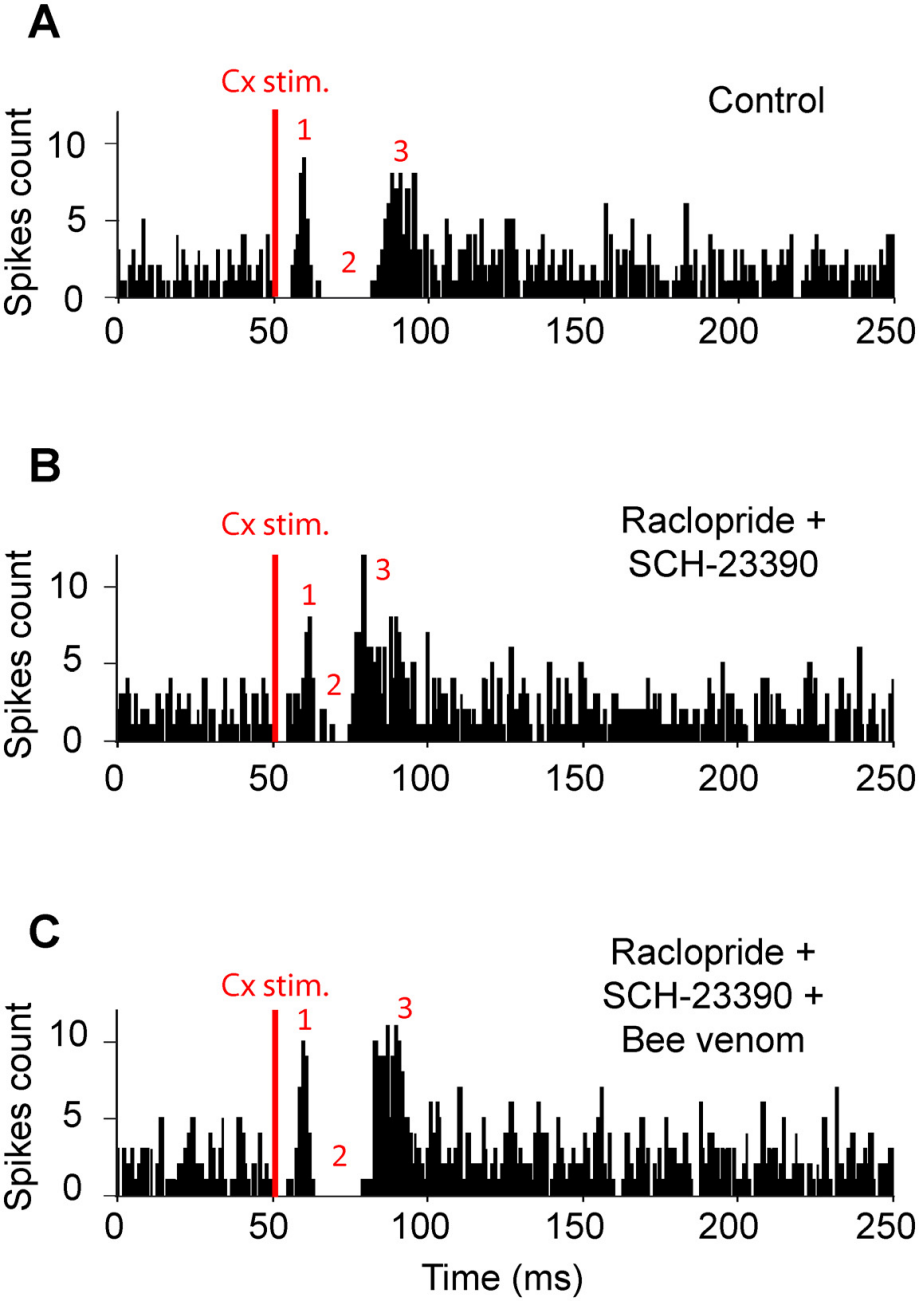
The effect of systemic injection of dopaminergic antagonists of D1- and D2-like receptors on the pattern of responses evoked by cortical stimulation in a SNr neuron is reversed by systemic injection of bee venom. (***A***) Classical triphasic excitatory-inhibitory-excitatory sequence evoked by stimulation of the orofacial sensori-motor cortex in control condition. (***B***) 25 minutes after injection of raclopride (1 mg/kg) plus SCH-23390 (0.5 mg/kg), the inhibitory component of the response of the same SNr neuron presented a marked reduction, whereas the late excitatory component was increased. (***C***) By 30 minutes following the systemic injection of BV, the inhibitory component of the cortically-evoked response was restored and the late excitatory component was decreased. Red numbers in ***A***-***C*** are as in Fig 4. The same number of cortical stimulations (red bar, n = 50) was applied in ***A-C***.

BV’s ability to reverse the alterations induced by the blockade of dopamine transmission was then tested using a combination of neuroleptics acting more rapidly than haloperidol (Fig 5). As previously described [34], simultaneous systemic injections of raclopride (2 mg/kg) and SCH-23390 (0.5 mg/kg) induced a rapid (< 10 min.), powerful and long lasting catalepsy paralleled with rapid change (< 20 min.) in SNr activity. This timing allowed for successive testing of the different conditions in the same neuron (n = 7): control (Fig 5A), neuroleptics (20–30 min after injection; Fig 5B) and neuroleptics+BV (injected 30 min after neuroleptics and recordings performed at least 30 min later; Fig 5C). The early excitation of the cortically-evoked responses was not significantly affected by raclopride+SCH23390. By contrast, the parameters of both the inhibition and the late excitation were markedly altered: inhibition duration was strongly shortened (-69.9% vs. control) and for the late excitation, latency was shortened (-17.7%) and duration was increased (+ 31.8%) while amplitude was unchanged. BV reversed the neuroleptic-induced alterations of both the inhibition and the late excitation, without modifying the unaffected early excitation (Table 2C).

### Bee venom impact on the spontaneous discharge and the cortically-evoked activity of SNr neurons in hemiparkinsonian rats

Animals with 6-OHDA lesion (n = 14) presented significant changes in the firing pattern of ipsilateral SNr neurons when compared to unlesioned animals (Table 1C)? This was evidenced by higher occurrence of bursts (*p* = 0.002, t-test) and increased percentage of spikes contributing to bursts (*p* = 0.012, t-test). 8 out of the 14 cells recorded were kept long enough to compare their firing properties before and 30 min after BV injection. No significant differences (Table 1D) were measured in the mean firing frequency (*p* = 0.097, paired t-test), recurrence of bursts (*p* = 0.182, paired t-test) and percentage of spikes contributing to bursts (*p* = 0.098, paired t-test).

**Fig 6:**
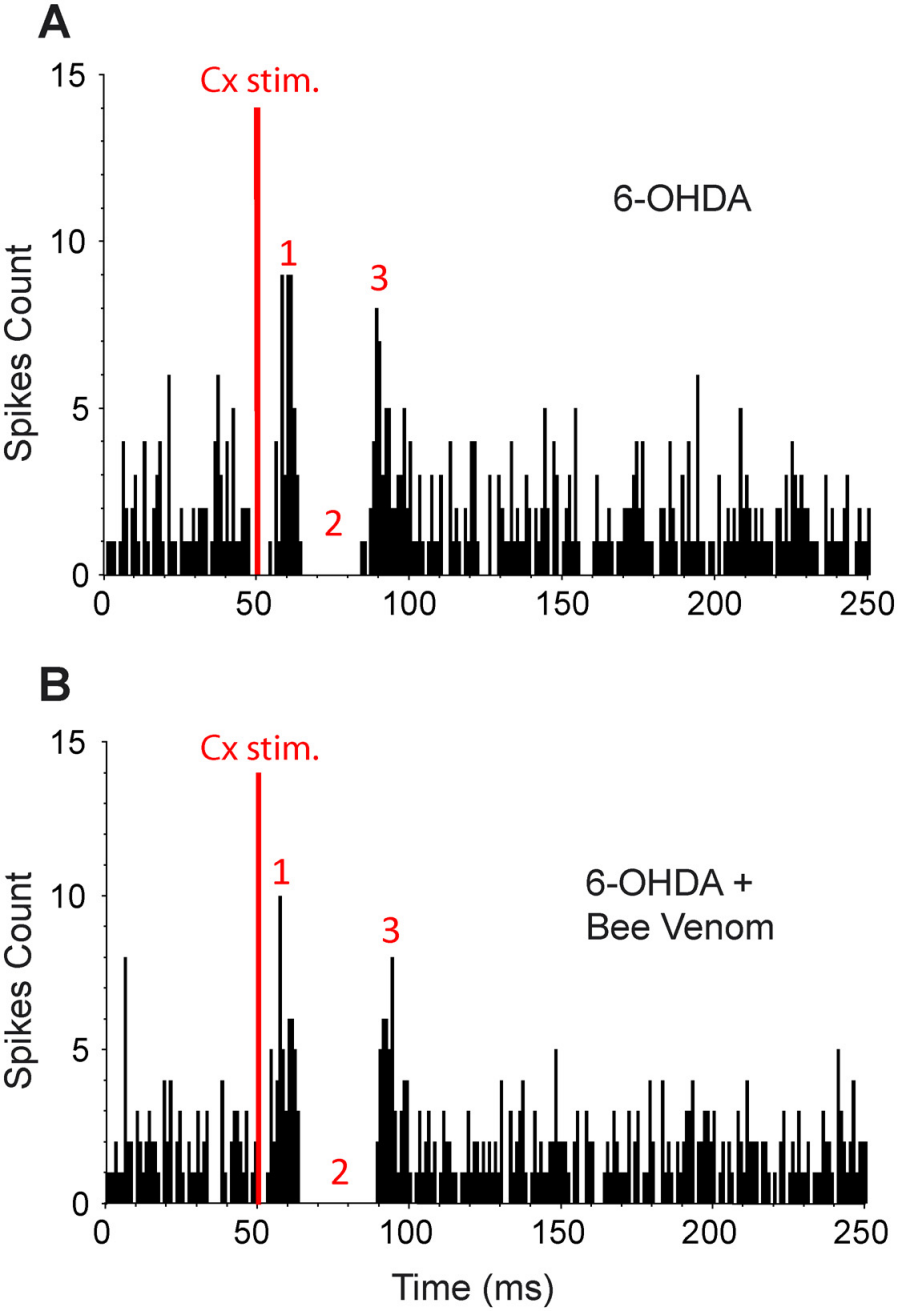
Effect of systemic injection of bee venom on the pattern of responses evoked by cortical stimulation in a SNr neuron from a 6-OHDA lesioned rat. (***A***) Triphasic excitatory-inhibitory-excitatory sequence evoked in a SNr neuron by stimulation of the orofacial sensori-motor cortex in the 6-OHDA condition. (***B***) 30 minutes after systemic injection of bee venom (3 μg/kg), the inhibitory component of the response of the same SNr neuron was markedly increased, whereas the early and late excitatory components were not modified. Red numbers in ***A***-***C*** are as in Fig 4. The same number of cortical stimulations (red bar, n = 70) was applied in ***A*** and ***B***.

The responses evoked by motor cortex stimulation were recorded in the same 8 SNr neurons from 6-OHDA animals, before and after BV injection (Table 2D and Fig 6). BV injection did not significantly affect the characteristics of the early and late excitations of the cortically-evoked responses. By contrast, the inhibition duration was significantly increased (+35.1%; p = 0.009, paired t-test). This suggests that BV acts selectively on the direct trans-striatal pathway in extensive dopamine lesion condition.

## Discussion

### Bee venom alleviates parkinsonian-like deficits

An antiparkinsonian effect of BV was first evidenced here in the haloperidol-induced catalepsy model, classically used to evaluate the anti-akinetic efficiency of potential antiparkinsonian compounds [37]. DA receptor blockade with a preferential D2 (haloperidol) or a combination of a D1 (SCH23390) and a D2 (raclopride) DA receptor antagonists, produces a high level of sedation mimicking aspects of parkinsonian akinesia. In this PD model, BV reversed haloperidol-induced catalepsy in a dose-dependent manner. Among the components that are involved in BV central actions, such as the mast cell degranulating peptide that binds voltage K+ channels [38] or melittin [39], apamin may likely contribute to the antiparkinsonian effect of BV. Indeed, apamin was previously reported to alleviate motor and non-motor PD deficits in lesional models [21] and was shown here to mimic the beneficial behavioral effects of BV injection in the neuroleptic-induced catalepsy model. Considering that apamin content represents about 2–3% of the BV mass [40,41], the efficient doses on parkinsonian symptoms of apamin are far higher than those of BV as recently also reported for their neuroprotective effects [13]. It could be that other BV components facilitate apamin central action by increasing the blood-brain barrier permeability and/or act synergically with apamin on common or different targets. The behavioral effects of apamin are classically ascribed to SK channel blockade. Apamin has been shown *in vitro* to antagonize the action of CyPPA, a positive gating modulator of SK channels which increases their apparent Ca^2+^-sensitivity by acting at the calmodulin binding domain [42]. Here we show that intracerebral CyPPA infusion efficiently blocks the effects of systemically-injected BV or apamin. Although the mechanisms of such interaction remain to be elucidated, it further supports the contribution of SK channels in the anti-cataleptic action of BV.

Motor-stimulant effect of apamin has been associated with increased activity of mesencephalic dopaminergic neurons and DA release in the striatum [21, 43]. Such action on dopamine neurons might contribute to the anti-cataleptic effect of BV as well as of apamin: increased dopamine tone could reduce the haloperidol-mediated blockade of striatal dopamine transmission at postsynaptic dopamine receptor level. However, action through modulation of dopamine neurons cannot account for the BV antiparkinsonian action evidenced here or for the reported beneficial effect of apamin [21] in the model of extensive lesion of nigrostriatal neurons. SK channels are not only expressed widely in dopaminergic neurons, but also in the overall basal ganglia network, including the main input station striatum, the subthalamic nucleus and the output structure SNr [44–46]. BV might then exert its symptomatic antiparkinsonian effects by acting not only on DA neurons when still present, but also on non-DA systems of the basal ganglia at advanced PD stage, presumably via the blockade of SK channels. Together with previous evidence for a neuroprotective effect of BV or apamin in PD models [9–14], the present findings suggest that this agent can combine symptomatic and disease-modifying properties by acting on different targets and pathological processes in PD state. It is noted that the anti-parkinsonian efficiency of BV treatment seems to be highly sensitive to the dose and the administration regimen, suggesting a rather narrow potential therapeutic window.

### Bee venom restores functional balance in the basal ganglia subcircuits

The site of action of BV in the cortico-basal ganglia-thalamo-cortical circuit is an important issue. To identify the cellular substrates of BV effect, we analyzed its impact on the activity of SNr neurons in the haloperidol-induced catalepsy and the 6-OHDA lesion models. Consistent with previous data obtained after injection of a mixture of D1 and D2 receptor antagonists [34], haloperidol-induced interruption of dopaminergic transmission dramatically affected the spontaneous discharge of SNr neurons, which switched from a tonic and regular to an irregular mode with high-frequency bursts and/or pauses. BV, at anti-cataleptic dose, did not regularize this abnormal pattern in SNr, thus contrasting with the normalizing impact of subthalamic nucleus high frequency stimulation, a powerful antiparkinsonian strategy [34,47]. Similarly, BV did not modify the spontaneous activity of SNr neurons in 6-OHDA-lesioned rats, which, consistent with previous reports, showed increased burst firing compared to control animals [48–50]. In contrast, BV impacted the cortically-evoked responses in SNr neurons in both neuroleptic and 6-OHDA PD models. As previously reported [27,51,52], cortical stimulation in naïve animals triggers a triphasic response in the SNr: i) an early excitation, involving the hyperdirect trans-subthalamic pathway, ii) an inhibition mediated by the trans-striatal direct pathway and iii) a late excitation attributed to the indirect trans-striatal pathway. Both haloperidol, a preferential antagonist of D2 dopamine receptors, and raclopride+SCH23390, which are respectively high affinity antagonists of D2- and D1-like receptors [53], increased the late excitation. This is attributable to the blockade of the inhibitory action exerted by dopamine through D2 receptors on the striatopallidal neurons at the origin of the indirect pathway. In support of this hypothesis, D2 receptor blockade by haloperidol or raclopride has been previously associated with increased striatal expression of preproenkephalin mRNA [54,55], a marker of the striatopallidal neurons, and with increased extracellular levels of GABA in the globus pallidus [56,57]. In addition, raclopride+SCH23390 also reduced the inhibitory component of the triphasic response [34], presumably due to SCH23390 action, blocking the excitatory influence exerted by dopamine on the D1 receptor-bearing neurons of the direct striatonigral pathway. All these alterations were either prevented or reversed by BV injection, indicating that BV restores the perturbed balance between the inhibitory and excitatory influences exerted by the trans-striatal direct and indirect pathways. BV had no major impact on transmission through the hyperdirect trans-subthalamic pathway, which was unaffected by haloperidol or raclopride+SCH23390. In 6-OHDA-lesioned animals, BV increased the inhibitory component of the triphasic response, whilst having no effect on the late excitation. These data suggest that BV, in dopamine-lesioned animals, acts mainly by strengthening the functional impact of the direct striatonigral pathway, which is reported as hypoactive in PD state, while being inefficient on the indirect pathway that is reported as overactive. Future investigations, involving intracerebral injections of BV or apamin, will allow further characterization of their action sites in the cortico-basal ganglia-thalamo-cortical circuits. In particular, previous evidence has been provided for a major contribution of SK channels to duration differences in the responses evoked by cortical stimulation in the direct and indirect striatal projection neurons[58]. Whether and how this determinant of striatal responses to cortical input is altered by dopamine denervation is a critical issue.

## Conclusions

This study provides experimental evidence for a symptomatic antiparkinsonian potential of BV and suggests that BV acts by counteracting imbalanced activity of the trans-striatal pathways. It reinforces the interest for BV raised by previous neuroprotection studies. The potential efficacy of BV on motor PD symptoms is currently under clinical trial (ClinicalTrials.gov identifier: NCT01341431).
